# Differences in Physical Activity, Sedentary Behavior, and Mental Health of the Older Population in South Korea Based on Marital Status and Gender

**DOI:** 10.3390/ijerph20031726

**Published:** 2023-01-18

**Authors:** Jeong-Hui Park, Tyler Prochnow, Christina Amo, Laurel Curran, Matthew Lee Smith

**Affiliations:** School of Public Health, Texas A&M Health Science Center, 212 Adriance Lab Rd., College Station, TX 77843, USA

**Keywords:** widowed adults, marriage, physical activity, gender, health behavioral factors

## Abstract

The primary purpose of the present study was to assess differences in physical activity (PA), sedentary behavior (SB), and mental health (i.e., depression, suicidal thoughts, and cognitive function) by marital status (i.e., married and widowed) within an aging population in South Korea. PA, SB, and mental health were evaluated in 9092 older adults by comparing the married group (*n* = 5773, 73.2 ± 5.9 years, 63.5%) to the widowed group (*n* = 3319, 75.8 ± 6.8 years, 36.5%). Between-group differences in PA, SB, depression, and cognitive function were tested using independent *t*-tests, and the association between marital status and gender was evaluated using two-way ANOVA. Suicidal thoughts were analyzed using a Mann–Whitney U-test. Older adults in the widowed group participated in significantly less PA (*p* < 0.001) and had higher SB (*p* < 0.001) per week, especially the widows, who had significantly less PA (*p* < 0.01) and had higher SB (*p* < 0.001) compared to married women. Participants in the widowed group experienced more depression (*p* < 0.001) and suicidal thoughts (*p* < 0.001) and had significantly lower cognitive function compared to participants in the married group (*p* < 0.001). Between the two groups, widowers were more vulnerable to all mental health factors compared to those in the married group. At the same time, widows were only more vulnerable to depression and cognitive function compared to women in the married group. Findings indicated that the presence of a spouse is strongly associated with higher PA levels, lower SB, and better mental health among older adults. Spouses are the primary social supports and play a major role in the health and emotional well-being of the aging population. Given the importance of the spouse, our study suggests that health educators working with older widows should prioritize several different forms of social support to benefit their physical and mental health.

## 1. Introduction

Rising life expectancy has led to an increase in the absolute number and proportion of the world’s population aged 65 and older. The United Nations (UN) has reported that older people account for more than one-fifth of the population in 17 countries, and it predicts that 155 countries will have an aging society by 2100, with the older population accounting for a majority (approximately 61%) of the world’s population [1]. The World Health Organization (WHO) classified Korea as an aging society in 2017, accounting for more than 14% of the population aged 65 or older [2]. Because a larger proportion of older adults have chronic conditions and physical and mental health needs, many countries may be underprepared or ill-equipped to provide healthcare and related services to their rapidly growing aging populations. As such, efforts are needed to better understand the aging Korean population’s current lifestyle behaviors and health needs and identify the impact of possible social supports and drivers. The United States (U.S.) Census Bureau also predicts that by 2060, 23% of the total population in the U.S. will be aged 65 or older, and 26.1% of the European society in 2050 will be over the age of 65 [3,4].

Although many older people wish to lead healthy and independent lives in older age, about 7% of older adults are affected by dementia (increasing from 1.7% in 60–65 years to 29.9% in 90–94 years), more than 20% experience depression and/or anxiety, and drug and/or alcohol misuse are also on a steady rise [5,6]. There are several causes for these issues, but the presence or absence of a spouse may be a primary factor [7,8]. A transition of marital statuses, such as the death of a spouse, is one of the most impactful events in one’s life. Because a spouse in older age is a primary source of social support and plays an essential role in their life [9], the death of a spouse (or loss of a close social tie) is strongly associated with premature mortality, morbidity, and poor quality of life. It can lead to increased social isolation [10,11,12]. Specifically, having a spouse at older ages encourages health-promoting behaviors and hinders health-compromising behaviors such as inactivity, unhealthy dietary habits, alcohol consumption, and nicotine use [13]. In addition, spousal connections have been known to be an important social factor in promoting physical activity (PA) [14].

Participating in regular PA and reducing sedentary behavior (SB) is associated with numerous good health outcomes and is also effective in preventing the risk of all-cause mortality such as obesity, diabetes, cardiovascular disease, and chronic diseases [15,16,17]. Several studies have demonstrated that strategies to increase PA and decrease SB among older adults can help improve their quality of life and reduce medical expenditures [18,19]. Old age is an important stage of life to improve the physical functions of daily life and delay the progression of diseases and disorders; however, most older people are at risk of leading an inactive lifestyle due to the natural progression of senescence. According to one study, only 14.7% of the older population in South Korea was found to meet the WHO’s recommended PA levels (i.e., at least 150 min of moderate-intensity PA per week or at least 75 min of vigorous-intensity PA per week, or a proportionate combining of moderate- to vigorous-intensity PA) [20].

Public health guidelines advise that participation in PA has many benefits to mental health and that maintaining regular participation in PA is important for mental well-being [21]. Especially, the complex experience of aging has been associated with adverse mental health experiences such as depression, suicidal behavior, and cognitive decline [22,23]. Previous research reports have shown that participating in PA reduces anxiety and depression [24], but, importantly, people aged 65 years and older are more vulnerable to depression compared to those under 65, partially due to physical inactivity. Another study found that people who reported experiencing suicidal ideation and attempted suicide recorded fewer daily steps compared to people who reported never experiencing either [25]. Furthermore, according to an eight-year cross-sectional study by Azevedo, there is an inverse relationship between PA, depression, and anxiety; higher PA was associated with lower symptoms at all middle-aged times, and frequent exposure to depression and anxiety predicted a decrease in PA levels [26]. Mental health vulnerability has also been reported to be closely related to cognitive function and possibly leads to dementia [27].

Guided by these factors, the primary aim of the present study was to investigate the differences in PA, SB, and mental health between older adults who are married and those who have lost a spouse. This loss can affect the physical and psychological health of the older population. Also, as a secondary aim, specific results were compared between genders for variables (i.e., PA, SB, and mental health) within the two groups, stratified according to marital status. This study contributes to the current literature by evaluating the role of close social connections (e.g., a spouse) on physical and psychological health among older adults in South Korea.

## 2. Methods

### 2.1. Study Design

The National Survey of Older Koreans is a series of nationwide cross-sectional surveys conducted by the Korea Institute for Health and Social Affairs and the Ministry of Health and Welfare in 2020 [28]. To obtain health-related information and data on the living conditions of the older population in South Korea, the survey is performed every three years. The survey is comprised of a total of 185 questions in 10 areas, including households (e.g., marital status, income levels, and education levels), health status, health behavior, functional status, and leisure and social activities. Stratified cluster sampling was used to sample the survey areas and household members, and all data were obtained through a questionnaire based on in-person interviews with professional surveyors.

### 2.2. Study Participants

This study analyzed 9092 of 10,097 older adults participating in the survey from 65 years to older. Participants who did not respond to questions related to the study’s variables (i.e., PA, SB, depression, suicidal thoughts, and cognitive function) or who were missing anthropometric information (e.g., height and weight) were excluded from analyses (*n* = 162). The present study also excluded responses when the intended subjects could not participate due to a disease and/or disorder (*n* = 233). The study sample was then divided into two groups of primary interest: those with a spouse (i.e., married group) and those who were widowed when they participated in the survey (i.e., widowed group). Groups were based on participant responses to the item, “Has a spouse (Married)” or “Bereaved”. Older people with other marital statuses at the time of the survey (i.e., single, divorced, and separated) were excluded from the study (*n* = 610) because the population was significantly smaller than the other two groups (i.e., married and widowed group). The sample size calculation assumed 80% power with alpha 0.05 and was calculated using G-Power software version 3.1.9 [29], and the adequate sample size was calculated to be at least 161 participants per group. Therefore, the sample size of this study satisfied the minimum sample size. The present study was approved by the Institutional Review Board of the Korea Institute for Health and Social Affairs (#2020-36).

### 2.3. Measures

#### 2.3.1. Anthropometric Characteristics

All participants’ height was measured by measuring tape with a trained research assistant, and weight was measured by a scale placed at the participants’ houses. If they did not have a scale, they were asked to fill in the most recently measured weight values. Weight was measured by rounding to the nearest 0.5 kg, and height was measured by rounding to the nearest 0.1 cm. Additionally, body mass index (BMI) was calculated by dividing the weight (kg) by the square of the height (m^2^).

#### 2.3.2. Physical Activity (PA)

Participants were asked to fill out free response questions such as, “How many days a week do you participate in PA?” and “On each of those days, how many minutes does your PA take?”. Responses were coded in numerical format. To quantify the PA participation time over the course of a week, the number of weekly PA bouts was multiplied by the daily minutes of PA [28].

#### 2.3.3. Sedentary Behavior (SB)

Time spent watching television has been used as a predictor of SB in previous studies, and people with higher television viewing time had lower levels of PA, such as walking, than those who do not [30,31,32]. Therefore, participants in this study reported the amount of time they were sedentary by responding to the question, ‘How many hours do you watch TV or listen to the radio on average per day?’. It was explained that participants should only report the time they watched TV or listened to the radio without paying attention to anything else, such as watching/listening while driving/commuting in transit. This was calculated by multiplying the number of SB times in a week by the minutes of SB time in a day.

#### 2.3.4. Mental Health

*Depression.* Depression was measured on a Short Form Geriatric Depression Scale (GDS) [33]. The GDS is a representative test widely used to measure and screen depression symptoms quickly, with measured reliability and validity (Cronbach’s alpha = 0.88, Coefficient = 0.66) [34]. Participants answered yes (1) or no (0) to a total of 15 questions (totals ranging from 0 to 15 points) related to depression, with higher scores indicating higher levels of depression. Scores of 7 points or less were considered to have no or minor depressive symptoms.

*Suicidal Thoughts.* To measure suicidal thoughts, participants were asked a dichotomous question “Have you ever thought of committing suicide after the age of 60?”. The questionnaire was used by translating some of the items in Attitude Toward Suicide (ATTS), developed by Renberg and Jacobsson [35]. The translated version of ATTS is useful for effectively and accurately evaluating the suicidal attitudes of Korean adults, which has been verified for reliability and validity (Cronbach’s alpha = 0.82) [36].

*Cognitive Function.* A Mini-Mental State Examination-Dementia Screening (MMSE-DS) was used to evaluate the cognitive function of older adults in this study. MMSE-DS is the most popular and universal test used to screen for dementia because of its high reliability and validity in screening patients with dementia syndrome (Coefficient = 0.88) [37]. MMSE-DS has eight areas including: time cognitive ability, space cognitive ability, memory perception, memory recall, attention focus and calculation, language ability, composition ability, understanding, and judgment. Each of the 30 questions was answered by yes (1) or no (0), with a maximum total score of 30 points. MMSE-DS, which was used to evaluate participants’ cognitive function, was classified as having a normal cognition level of 25 points or higher, mild dementia from 20 to 24 points, moderate dementia from 13 to 20 points, and severe dementia below 12 points [37].

### 2.4. Statistical Analysis

The demographic information (i.e., gender, age, and education level) and anthropometric information (i.e., height, weight, and body mass index) were analyzed by descriptive statistics (means and standard deviations) and independent *t*-tests in SPSS 25.0 version (IBM, Chicago, IL, USA). To ascertain between-group (i.e., married group and widowed group) differences in PA, SB, depression, and cognitive function, the current study was tested through independent *t*-tests. The association between marital status and gender was evaluated by a two-way analysis of variance (ANOVA). Suicidal thoughts were analyzed by Mann–Whitney U-test because the suicidal thoughts variable was measured by a categorical variable (i.e., yes or no). All statistical significance was determined at *p* < 0.05.

## 3. Results

Table 1 summarizes sample information (i.e., demographic, socioeconomic, and anthropometric) within the married group (*n* = 5773) and widowed group (*n* = 3319). The average age of participants in the two groups was 70 years or older (married: 73.2 ± 5.9 and widowed: 75.8 ± 6.8). The married group was mostly men (95.2%), and the widowed group was mostly women (82.7%). On average, the married group was taller and weighed more than the widowed group. Although there was a significant difference in height (*p* < 0.001) and weight (*p* < 0.001) between the groups, there was no significant difference in body mass index (*p* > 0.05). The education level of the older population in the two groups was below middle school graduation. Finally, 53.6% of the participants in the married group graduated from elementary school, and 36.1% of the participants graduated from middle school. Most of the participants in the widowed group indicated below elementary school graduation as the level of education (83.5%).

Table 2 presents the comparison of average PA, SB, and mental health (i.e., depression, suicidal thoughts, and cognitive function) between the married group and the widowed group. Results suggested that individuals in the widowed group were less active per week (married: 134.5 ± 180.4 min; widowed: 103.2 ± 151.7 min, *p* < 0.001) and reported more sedentary behavior per week (married: 31.1 ± 15.4 h; widowed: 27.2 ± 13.2 h, *p* < 0.001) than those in the married group. Levels of reported depressive symptoms were significantly higher in the widowed group than in the married group (4.1 ± 3.7 points vs. 2.9 ± 3.1 points; *p* < 0.001). Although reported suicidal ideation was modest, the widowed group accounted for a significantly higher proportion than those in the married group (2.5% vs. 1.3%; *p* < 0.001). The cognitive function scores in the married group were higher than those in the widowed group (25.0 points vs. 23.1 points; *p* < 0.001). 

**Figure 1:**
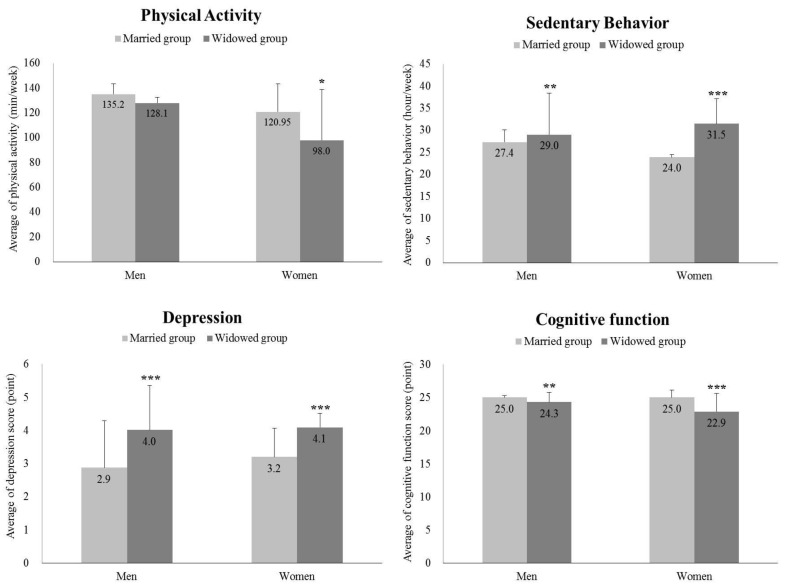
The comparisons of participation time in physical activity and sedentary behavior and score of depression and cognitive function in gender and groups. *** *p* < 0.001, ** *p* < 0.01, * *p* < 0.05.

Figure 1 presents comparisons of PA, SB, depression, and cognitive function between the married and widowed groups by gender. When differentiating by gender, men in the widowed group reported fewer minutes of weekly PA than those in the married group (married: 135.2; widowed: 128.1), but there was no significant difference (*p* > 0.05). Unlike men’s results in PA, women’s PA between the two groups showed a significant difference (married: 121.0; widowed: 98.0; *p* < 0.05). Widowed men reported sitting longer each week than married men (married: 27.4; widowed: 29.0; *p* < 0.01). Widowed women also reported sitting longer than married women (married: 24.0; widowed: 31.5; *p* < 0.001). Furthermore, the widowed men had higher depression scores than married men (married: 2.9; widowed: 4.0; *p* < 0.001), and widowed women’s depression scores were consistent with the men’s outcome (married: 3.2; widowed: 4.1; *p* < 0.001). Cognitive function scores were found to be higher for both men (married: 25.0; widowed: 24.3; *p* < 0.01) and women (married: 25.0; widowed: 22.9; *p* < 0.001) in the married group than for those in the widowed group. 

## 4. Discussion

The current study aimed to examine the differences in PA, SB, and mental health, between older adults who are married and those who have lost a spouse. The transition from having a spouse to becoming a widow/widower represents the loss of close social ties and is often a life-defining event [11]. Thus, it is essential to understand the impact of marital status on the lives and health of older adults to better provide for their welfare. Further, this study supplements the outcomes of previous studies [13,14,15,20] by providing implications for improving new social connections and activity behaviors for older people who have faced a marital status transition.

The transition of marital status in older age demonstrated a significant association with PA, SB, and mental health in this study. In the present study, older adults’ PA in the widowed group was approximately 30 min less per week than their married counterparts, and older adults in the widowed group spent more than 4 h per week sitting compared to those in the married group. These results are consistent with previous studies. Robins et al. [38] found that older people living with a partner/spouse reported better general health and higher levels of PA than those who did not. Additionally, Fingerman et al. [39] suggested that both men and women with a spouse were less likely to watch television for more than 3 h a week compared to those living alone. A possible explanation for the significant association between widowed older adults and poor health behaviors (low PA and high SB) might be the absence of spouses (one of the primary factors in social support and/or ties in older age), the changing of roles within family and social networks, or the loss of social connections (which temporarily and/or permanently led to social isolation). Parallel literature supports the possibility of the association between negative influences on older adults’ health and high levels of social isolation (i.e., being widowed and the loss or death of family or friends) while aging [40,41,42]. 

Therefore, understanding that the transition to marital status in older age can isolate them from society can help in preventing their poor health behaviors (reducing PA and increasing SB). As seen in results presented here and elsewhere, it is vital for interventions to engage older adults who have lost spouses to maintain or improve PA while providing social support. Thus, PA intervention studies have also aimed to impact social isolation, loneliness, or low social support in older adults [43]. Within a review of such interventions, results indicated a small significant positive effect for PA interventions with the strongest effects obtained in diseased populations and group exercise settings [43]. However, the effect of PA on psychosocial outcomes in older adults was found to be inconsistent [43].

Each factor of mental health (i.e., depression, suicidal thoughts, and cognitive function) pointed out that participants in the widowed group had worse mental health than those in the married group. The most interesting observation in mental health between the two groups was the cognitive function score. Prior studies have reported that decreased cognitive function among older adults is influenced by lifestyle factors and brain stimulation, including lack of PA and social isolation [44] and unhealthy diets [45,46]. Specifically, in the study presented here, the average cognitive function score of the people in the widowed group was 23.1 points on average, and they were likely to be diagnosed with mild dementia [37]. However, the average cognitive function score of people in the married group was normal, with an average of 25.0 points [37]. Therefore, the low cognitive function of the older adults in the widowed group needs to be examined in a multilateral effort. In addition, this study’s outcomes suggest that the spouse’s death may make the remaining spouse more socially isolated, which is related to low PA and high SB. Unfortunately, these factors further compound and increase the risk of mortality as a meta-analysis indicated social isolation odds ratio (OR) = 1.29, loneliness OR = 1.26, and living alone OR = 1.32, corresponding to an average of 29%, 26%, and 32% increased likelihood of mortality, respectively [47]. Further, social isolation and PA also impact the quality of life and cognitive functioning in older adults as well [43,48].

Lifestyle changes due to the transition of marital status in later life may be a sign of increasing social isolation for widows rather than widowers. Although men and women in the widowed group showed lower PA, higher SB, and lower mental health than men and women in the married group, the study found that widows were more vulnerable to all PA, SB, and mental health variables compared to widowers. These results are consistent with previous studies that reported that widows are more likely to have days of restricted activity and stay in bed longer than married women, unlike married men and widowers [49]. This is because older widows are more likely to experience mobility-related disabilities and concerns related to functional restrictions [50]. This may have a more negative impact on their health behaviors, such as PA, SB, and mental health in later life than it would for widowers. In addition, it cannot be ignored that widowed men can still have the same social status and resources available as married men in patriarchal countries such as Asia [51]. 

As social ties decrease in older age, methods for social support need to be prioritized to enable widows to avoid social isolation and participate in PA. One way to do so is by mobilizing social networks such as family, neighborhood, and community resources [48]. Specifically, a study on social support and social networks among a sample of older Canadian adults indicated a need for diverse forms of social participation, social support, and social connections outside of the traditional close friends and family members [52]. Further studies have suggested several avenues to improve and expand social integration and social support networks through guided cognitive reframing [53], personal identity and resilience assistance training [54], and creating a connection plan similar to other PA, functioning, or nutrition plans created for individuals [55]. 

The present study has the following strengths and limitations. The study contained 9092 older participants, which was adequate for generalizability in Korea. The study’s main findings add to the existing literature about the association between the older population and PA, SB, and mental health in addition to providing new insights regarding factors influenced by the transition of marital status. Nevertheless, since all variables were measured by self-report questionnaires, the study could not exclude that the values of PA and SB may have been over- or under-estimated. Although PA was an important variable in this study, this study only examined the frequency and duration, not intensity and types. Further study is necessary to measure the PA and SB of the older population using strong monitoring devices such as accelerometers to compensate for these limitations. Since the majority of the participants in the married group were male while the majority of the participants in the widow group were female, the gender proportion between the two groups should be considered when formulating future studies. Additionally, other studies in the future need to demonstrate more evidence with various countries and races, because participants in this study were limited to older Korean adults.

## 5. Conclusions

The loss of a spouse in older age may be one of the factors that exacerbate physical and mental health outcomes. More specifically, the change in social network and social support through the loss of a spouse can have dramatic effects across the health behavior spectrum. Therefore, health educators and/or practitioners may wish to focus on social support for widowed older adults. Various interventions that can promote PA [56,57] and reduce SB to restore the temporary/permanent disconnections of widowed adults’ closest social connections can improve their social connectedness and activity behavior. In addition, it can be suggested that supporting widowed older adults to form new social connections can positively affect their physical and mental functions.

## Figures and Tables

**Table 1:** Participants’ characteristics and anthropometrics information.

Variable		Married Group (*n* = 5773)		Widowed Group (*n* = 3319)	
		No. (%)	Mean ± SD	No. (%)	Mean ± SD
Age (year)			73.2 ± 5.9		75.8 ± 6.8
Gender	Men	5495 (95.2)		574 (17.3)	
	Women	278 (4.8)		2745 (82.7)	
Anthropometrics	Height (cm)		163.3 ± 8.3		157.1 ± 7.3 ***
	Weight (kg)		63.0 ± 8.4		58.2 ± 8.3 ***
	BMI (kg·m^−2^)		23.6 ± 2.4		23.6 ± 2.6
Education	≤Elementary School	3094 (53.6)		2769 (83.5)	
	≤Middle School	2085 (36.1)		495 (14.9)	
	≤High School	210 (3.6)		24 (0.7)	
	≥Undergraduate	384 (6.7)		31 (0.9)	

SD: Standard Deviation, BMI: Body Mass Index; *** *p* < 0.001; Education: educational status (for example, ≤elementary school represents individuals who achieved only up to elementary school education).

**Table 2:** Comparison of physical activity, sedentary behavior, and mental health between the married and widowed group.

Variable	Married Group (*n* = 5773)		Widowed Group (*n* = 3319)		95% Confidence Intervals	
	No. (%)	Mean ± SD	No. (%)	Mean ± SD	Lower	Upper
Physical activity (min/week)		134.5 ± 180.4		103.2 ± 151.7 ***	23.996	38.552
Sedentary behavior (h/week)		27.2 ± 13.2		31.1 ± 15.4 ***	−4.497	−3.296
Depression scores (point)		2.9 ± 3.1		4.1 ± 3.7 ***	−1.324	−1.04
Suicidal thoughts	76 (1.3)		83 (2.5) ***			
Cognitive function scores (point)		25.0 ± 5.1		23.1 ± 5.3 ***	1.672	2.118

SD: Standard Deviation, *** *p* < 0.001; An adequate sample size was calculated to be at least 161 participants per group, and the sample size of this study satisfied the minimum sample size.

## Data Availability

The datasets used and/or analyzed during the current study are available from the corresponding author upon reasonable request.
